# Recombinant Sheep Pox Virus Proteins Elicit Neutralizing Antibodies

**DOI:** 10.3390/v8060159

**Published:** 2016-06-07

**Authors:** Olga V. Chervyakova, Valentin L. Zaitsev, Bulat K. Iskakov, Elmira T. Tailakova, Vitaliy M. Strochkov, Kulyaisan T. Sultankulova, Nurlan T. Sandybayev, Gulshan E. Stanbekova, Daniyar K. Beisenov, Yergali O. Abduraimov, Muratbay Mambetaliyev, Abylay R. Sansyzbay, Natalia Y. Kovalskaya, Lev. G. Nemchinov, Rosemarie W. Hammond

**Affiliations:** 1 Research Institute for Biological Safety Problems, RK ME&S – Science Committee, Gvardeiskiy 080409, Kazakhstan; ovch@mail.ru (O.V.C.); biosafety@biosafety.kz (V.L.Z.); tailakova_86@mail.ru (E.T.T.); vstrochkov@biosafety.kz (V.M.S.); sultankul70@mail.ru (K.T.S.); nurlan.s@mail.ru (N.T.S.); yergali.a@gmail.com (Y.O.A.); murat@biosafety.kz (M.M.); sansyzbai-ar@biosafety.kz (A.R.S.); 2 M. A. Aitkhozhin’s Institute of Molecular Biology and Biochemistry, RK ME&S – Science Committee, Almaty 050012, Kazakhstan; bulat.isakakov@mail.ru (B.K.I.); gulshanst@yahoo.com (G.E.S.); daniyar.b@mail.ru (D.K.B.); 3 United States Department of Agriculture, Agricultural Research Service, Molecular Plant Pathology Laboratory, Beltsville, MD 20705, USA; natalia.kovalksaya@ars.usda.gov (N.Y.K); lev.nemchinov@ars.usda.gov (L.G.N.)

**Keywords:** sheep pox virus, vaccinia virus, recombinant protein, prokaryotic expression, neutralizing antibodies, immunogenicity

## Abstract

The aim of this work was to evaluate the immunogenicity and neutralizing activity of sheep pox virus (SPPV; genus *Capripoxvirus*, family *Poxviridae*) structural proteins as candidate subunit vaccines to control sheep pox disease. SPPV structural proteins were identified by sequence homology with proteins of vaccinia virus (VACV) strain Copenhagen. Four SPPV proteins (SPPV-ORF 060, SPPV-ORF 095, SPPV-ORF 117, and SPPV-ORF 122), orthologs of immunodominant L1, A4, A27, and A33 VACV proteins, respectively, were produced in *Escherichia coli*. Western blot analysis revealed the antigenic and immunogenic properties of SPPV-060, SPPV-095, SPPV-117 and SPPV-122 proteins when injected with adjuvant into experimental rabbits. Virus-neutralizing activity against SPPV in lamb kidney cell culture was detected for polyclonal antisera raised to SPPV-060, SPPV-117, and SPPV-122 proteins. To our knowledge, this is the first report demonstrating the virus-neutralizing activities of antisera raised to SPPV-060, SPPV-117, and SPPV-122 proteins.

## 1. Introduction

Sheep pox is a highly contagious viral disease of small ruminants that is endemic in the Near East and Central Asia, India, China, and Central and Northern Africa. The disease causes significant economic losses by reduced hide and wool quality, the prevention of the importation of new breeds of sheep into endemic regions, and other production losses in animal husbandry worldwide [1,2,3,4]. The agent of sheep pox disease is sheep pox virus (SPPV), the type species of the *Capripoxvirus* genus, subfamily *Chordopoxvirinae*, family *Poxviridae*. In addition to SPPV, the capripoxviruses include goatpox virus (GTPV) and lumpy skin disease virus, all of which cause pox skin lesions, are of great economic importance, and are potentially emerging disease threats [5]. To control the spread of sheep pox infection, attenuated virus vaccines are widely used in the Republic of Kazakhstan and other Former Soviet Union countries [6,7,8]. In spite of the high protection afforded by live vaccines, the possible presence of residual virulence in the latter restricts their use in disease-free areas and has led to the exploration of new approaches in vaccine development involving recombinant DNA technology [9,10,11,12,13,14,15,16,17].

SPPV is a large (>200 nm), enveloped virus containing a linear double-stranded, non-fragmented DNA genome of ~150 kb encoding at least 147 putative genes [18]. The two major infectious forms of poxviruses are the intracellular mature virion (IMV) and extracellular enveloped virion (EEV). IMVs are assembled in the cytoplasm and are composed of a core particle containing the genome and numerous enveloped enzymes. At least 11 proteins are included in the envelope of IMVs [19,20,21,22,23,24,25,26,27]. As IMVs accumulate, they may be freed from the cell by apoptosis or alternatively can be wrapped in additional trans-Golgi-derived membranes and released from infected cells as EEV. The lipoprotein membrane of EEV contains several unique glycosylated and non-glycosylated proteins involved in virus entry [28,29,30,31,32,33,34]. 

The complex structure and the presence of a large number of proteins in virions presents challenges to the identification of immunomodulatory proteins of pox viruses. At least 12 immunogenic proteins have been detected in SPPV [35,36,37]. Immunogenicity of soluble antigens was confirmed experimentally in a study where blood serum obtained after immunization of animals with soluble antigens of SPPV neutralized virus infectivity in cell culture and protected experimental animals against infection [38]. Moreover, several antigenic proteins, and their corresponding genes, have been identified and used in antibody detection of SPPV in blood sera of sheep, goats, and cattle by enzyme-linked immunosorbent assay (ELISA) [39,40,41,42].

To date there is lack of data regarding the immunological properties of individual SPPV proteins and the neutralizing activity of antibodies raised to these proteins. In our study, we cloned the genes encoding the structural proteins SPPV-060 and SPPV-117 (IMV envelope components), SPPV-122 (EEV envelope component), and SPPV-095 (found in the core of virion), produced the protein in a prokaryotic expression system, and evaluated their antigenicity and immunogenicity, along with virus neutralizing activity of antibodies raised to these proteins in cell culture.

## 2. Materials and Methods

### 2.1. Viruses

The SPPV strain “NISKhI” was kindly provided by the Microbial Collection of the Research Institute for Biological Safety Problems RK ME&S – Science Committee, Gvardeiskiy 080409, Kazakhstan. For SPPV cultivation, a primary lamb kidney cell line maintained by the Laboratory of Cellular Biotechnology of the Research Institute for Biological Safety Problems RK ME&S – Science Committee was used.

### 2.2. Cloning of SPPV Genes

Genes *SPPV-060* (GenBank ID: NP_659632), *SPPV-095* (GenBank ID: NP_659667), *SPPV-117* (GenBank ID: NP_659689), and *SPPV-122* (GenBank ID: NP_659694) were PCR-amplified from viral DNA, extracted from SPPV virions with Trizol (Invitrogen, Carlsbad, CA, USA), using the primer pairs described in Table 1 (restriction sites are underlined, start codon is indicated in italics). The amplified products were digested with respective enzymes (Table 1). Restriction fragments were cloned into the plasmid vectors pET23c and pET26b (containing a HIS_6_-tag on the C-terminus), and giving rise to pET23c/*SPPV-060*, pET26b/*SPPV-095*, pET26b/*SPPV-117* and pET26b/*SPPV-122*. *SPPV-060* (*SPPV-060∆TM*; N (1) – C (188)) and *SPPV-122* (*SPPV-122∆TM*; N (68) – C (195)) genes were constructed to express only the extravirion domains of their encoded proteins in order to increase protein expression. The transmembrane domains were identified using the software TMHMM Server (Version 2.0) [43]. Comparative analysis of amino acid sequences of the cloned proteins and their orthologs was performed using BLAST software [44]. pET vectors were obtained from EMD Biosciences Inc. (Gibbstown, NJ, USA).

Amplification reactions were performed in 50 µL containing 5 µL 10× PCR buffer (Qiagen, Valencia, CA, USA), 1 µL 10 mM dNTPs (NEB, Ipswich, MA, USA), 0.1 µL template DNA (100 ng/µL), 1 µL of each primer (20 pmol/µL), and 0.25 µL Taq DNA polymerase (1.25 Units, Qiagen). The amplification conditions for *SPPV-122* were 94 °C 5 min, followed by 30 cycles of 94 °C 1 min, 45 °C 1 min, 72 °C 1 min, and a final extension at 72 °C 7 min. The annealing temperature for amplification reactions of *SPPV-060*, *SPPV-095*, and *SPPV-117* was 50 °C. All plasmids were sequenced to verify the integrity of the inserts.

### 2.3. Gene Expression, Protein Extraction, Purification, and Raising Specific Antibodies

Plasmids pET23c/*SPPV-060*, pET26b/*SPPV-095*, pET26b/*SPPV-117*, and pET26b/*SPPV-122* were transformed into *Escherichia coli* strain BL21 (DE3) (Stratagene, La Jolla, CA, USA) or strain T7 (NEB, Ipswich, MA, USA) according to the manufacturer’s instructions. Bacterial cells were grown in 50 mL LB-kan (Luria-Bertani broth, containing 50 µg/mL of kanamycin) or LB-amp (LB broth containing 50 µg/mL of ampicillin) at 37 °C to a cell density of OD600 = 0.6–1.0. Gene expression was induced by addition of IPTG to final concentration 1 mM to the bacterial suspension with subsequent incubation for 2 h at 37 °C. The cells were harvested by centrifugation at 5000× *g* for 15 min and the bacterial pellet was stored at −70 °C until protein extraction.

To prepare SPPV-060 and SPPV-117 protein samples for specific antibody production, the bacterial cell pellet was resuspended in 5 mL of 1× Laemmli sample buffer (Bio-Rad, Hercules, CA, USA) supplemented with β-mercaptoethanol (2.5% *v*/*v*) and boiled for 10 min. The resulting samples were separated by electrophoresis in a Novex 10%–20% Tris-Glycine Gel (Novex, Carlsbad, CA, USA) and visualized by staining with Simply Blue Safe Stain (Invitrogen, Carlsbad, CA, USA). Protein bands corresponding to the recombinant SPPV proteins SPPV-060 and SPPV-117 were excised from the gel and sent to the Pacific Immunology Corp. (Ramona, CA, USA) for rabbit polyclonal antibody production. Four-fold immunization was conducted at intervals of three weeks. The first immunization was performed by administration of the protein with complete Freund’s Adjuvant; proteins with incomplete Freund’s Adjuvant were used for the next three injections. One week after the last immunization, the animals were bled to obtain the antiserum.

Inclusion bodies (IBs) formed in *E. coli* producing SPPV-095 and SPPV-122 proteins were purified using the BugBuster Master Mix Protein Extraction Reagent (Novagen, Madison, WI, USA), resuspended in the same reagent (according manufacturer’s instructions) and used for rabbit polyclonal antibody production at the Research Institute for Biological Safety Problems RK ME&S Science Committee. The animals were immunized twice at an interval of two weeks. The first injection was conducted with purified IBs, and the second injection was performed with a mixture of IBs and Montanide ISA-70 (Seppic, Puteaux, France). Three weeks after the second immunization, the animals were bled to obtain antiserum.

### 2.4. Western Blot Analysis

For immunodetection, recombinant proteins were separated in a Novex 10%–20% Tris-Glycine Gel (Novex, Carlsbad, CA, USA), transferred to a nitrocellulose membrane (Invitrogen, Carlsbad, CA, USA) and probed, according to the manufacturer`s instructions, with either the anti-His (C-term)-AP antibody (Invitrogen, Carlsbad, CA, USA), antigen-specific antiserum produced in rabbits, or antiserum obtained from sheep experimentally infected with SPPV using a dilution of 1:1000. After protein transfer, the nitrocellulose membrane was briefly incubated for 1 h at room temperature (RT) or overnight (ON) at 4 °C in blocking buffer (1× PBS, containing 5% non-fat dry milk). The membrane was probed with primary antigen-specific serum for two hours at RT or ON at 4 °C, washed three times with PBS-Tween 20 (1× PBS supplemented with 0.1% (*v*/*v*) Tween 20) and incubated in alkaline phosphatase-conjugated goat anti-rabbit or anti-sheep antibody for 1–2 h at RT. After three washes with PBS-Tween 20, the membrane was developed by utilizing the BCIP/NBT Phosphatase Substrate System (Kirkegaard and Perry, Inc., Gaithersburg, MD, USA), according to the manufacturer’s instructions.

### 2.5. Virus Neutralization Studies

A primary lamb kidney cell line was used to perform virus neutralization tests. The confluent monolayer culture was trypsinized and resuspended in complete medium. Cells were counted using a hemocytometer and 5 × 10^4^ cells/well (0.2 mL/well final) in a 96-well tissue culture plate. Plates were incubated until the cells were confluent.

The sera under study were inactivated by heating at 60 °C for 30 min and diluted two-fold starting from 1:2 to 1:64, preparing 2 mL of each dilution and using the supporting cell maintenance medium of primary lamb kidney cell cultures as the diluent. Each dilution of serum was added to an equal volume of SPPV in a selected dose, e.g., 100 tissue cytopathic doses (50% tissue culture infectious dose (TCID50)). Mixtures of virus with the serum dilutions were incubated at 4 °C for 16 h. Two hundred µL of the mixture were added to each of four wells containing the confluent monolayer cell cultures in a 96-well plate (Corning Inc., Corning, NY 14831, USA). The cell cultures were incubated for one week at 37 °C to observe cytopathic effects (CPE). The results were read on the seventh day of incubation and were based on the presence of SPPV-induced CPE. The dilution that inhibits CPE caused by virus in 50% of infected cell cultures is considered to be the antibody titer of the serum under study. The following controls were used: virus control (virus + medium); serum control (serum + medium), and control-mock (medium).

### 2.6. Light Microscopy

96-well plates containing cell cultures with different treatments were examined using a Leitz diavert microscope with a CAMV200 digital camera and Motic Images 2000 software (Version 1.3).

## 3. Results

### 3.1. Gene Expression and Purification of Recombinant Proteins

**Figure 1:**
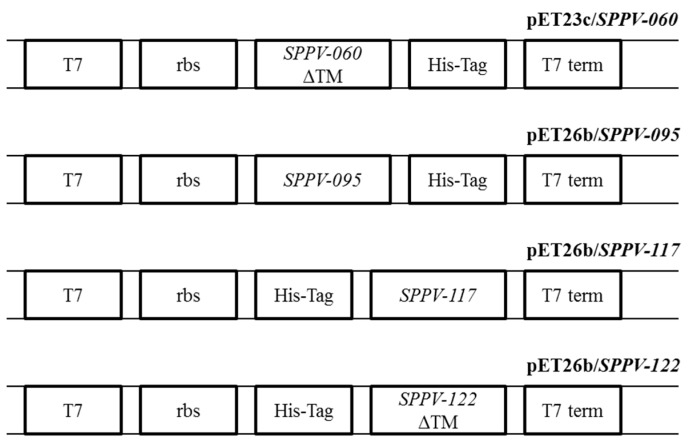
Schematic representation of pET-based expression cassettes encoding sheep pox virus (SPPV) proteins. Bacteriophage T7 promoter (T7); ribosomal binding site sequence (rbs); SPPV-060, SPPV-095, SPPV-117, SPPV-122 (SPPV gene sequences); deleted transmembrane domain (ΔTM); sequence coding six histidine molecules (His-Tag); T7 terminator (T7 term).

**Figure 2:**
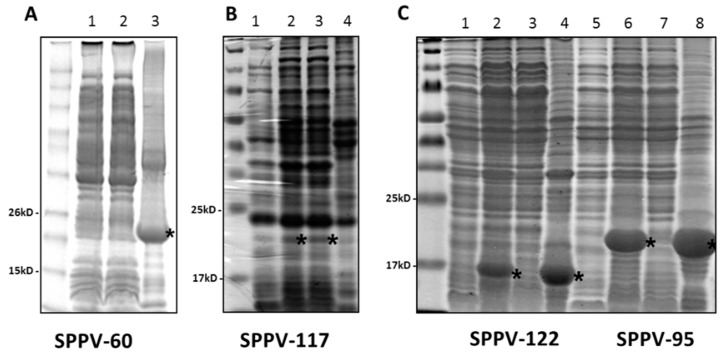
Denaturing 10%–20% polyacrylamide gel electrophoretic analysis of the protein fractions produced in E. coli transformed by pET23c/SPPV-060, pET26b/ SPPV-117, pET26b/SPPV-122 and pET26b/SPPV-095. (**A**) SPPV-060 protein analysis. Lane 1: 5 µL of total protein fraction; Lane 2: 5 µL of soluble protein fraction; Lane 3: 10 µL of protein fraction after IB purification. The gel was stained with SimplyBlue SafeStain (Invitrogen, Carlsbad, CA, USA). M: BenchMark™ Pre-Stained Protein Ladder (Invitrogen, Carlsbad, CA, USA). (**B**) SPPV-117 protein analysis. Lane 1: 5 µL of total proteins prior to IPTG induction; Lane 2: 5 µL of total protein fraction; lane 3: 5 µL of soluble protein fraction; lane 4: 10 µL of protein fraction after IB purification*.* The gel was stained with Coomassie Brilliant Blue R-250. M: Spectra Multicolor Broad Range Protein Ladder (Thermo Scientific, Waltham, MA, USA). (**C**) SPPV-122 protein (Lanes 1–4) and SPPV-095 protein (Lanes 5–8) analysis. Lanes 1, 5: 5 µL of total protein prior to IPTG induction; Lanes 2, 6: 5 µL of total protein fractions; Lanes 3, 7: 5 µL of soluble protein fractions; Lanes 4, 8: 10 µL of protein fraction after IB purification. The gel was stained with Coomassie Brilliant Blue R-250. M: Spectra Multicolor Broad Range Protein Ladder (Thermo Scientific, Waltham, MA, USA). The recombinant SPPV proteins are indicated by small asterisks.

To obtain antiserum specific to SPPV proteins for further immunological investigations, the genes encoding these proteins were amplified from the virus and inserted into pET-based expression vectors (Figure 1). Constructs containing the SPPV-060 and SPPV-122 genes were engineered to express only extravirion domains in order to enhance protein production in bacterial cells. Induction of recombinant protein expression in bacteria using IPTG resulted in the production of SPPV-060 (22 kDa; Figure 2A, lane 3), SPPV-095 (20 kDa; Figure 2C, lane 6), SPPV-117 (19 kDa; Figure 2B, lane 2), and SPPV-122 (16 kDa; Figure 2C, lane 2) proteins. The sizes of these proteins were consistent with the predicted molecular masses. Electrophoretic analysis revealed that the SPPV-117 protein was produced in a soluble form (Figure 2B, lane 3), whereas SPPV-060 (Figure 2A, lane 3), SPPV-095 (Figure 2C, lane 8), and SPPV-122 (Figure 2C, lane 4) proteins were localized in IBs. The purified recombinant proteins were used for rabbit polyclonal antibody production (see Materials and Methods).

### 3.2. Immunodetection of Recombinant Proteins

**Figure 3:**
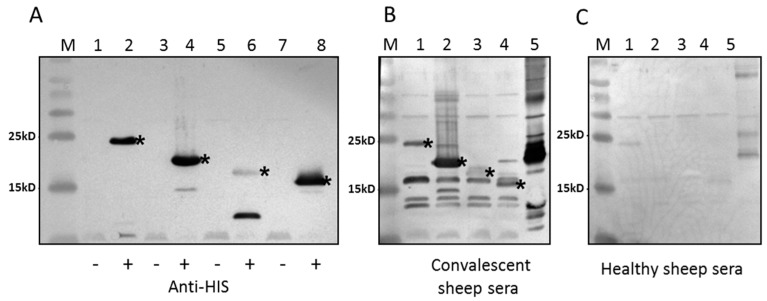
Western blot analysis of the SPPV recombinant proteins produced in *E.coli*. (**A**) Protein gel loaded with 5 µL of total protein fractions before (Lanes 1, 3, 5, 7) and after IPTG-induction (Lanes 2, 4, 6, 8). Lanes 1, 2: SPPV-60; Lanes 3, 4: SPPV-095; Lanes 5, 6: SPPV-117; Lanes 7, 8: SPPV-122. Anti-His (C-term)-AP antibody (Invitrogen, Carlsbad, CA, USA) was used to develop the blot. (**B**, **C**) Protein gel loaded with 5 µL of total protein fractions after induction of SPPV-060 (Lane 1), SPPV-095 (Lane 2), SPPV-117 (Lane 3), SPPV-122 (Lane 4), and purified sheep pox virus (Lane 5). The sera from the sheep experimentally infected with the sheep pox virus (**B**) and from healthy sheep (**C**) were used. M: Spectra Multicolor Broad Range Protein Ladder (Thermo Scientific, Waltham, MA, USA). The recombinant SPPV proteins are indicated by asterisks.

**Figure 4:**
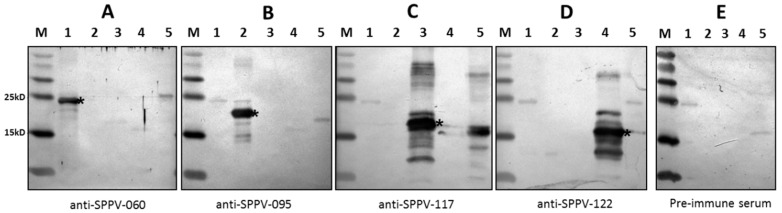
Western blot analysis of the recombinant SPPV proteins produced in *E.coli*. Protein gels loaded with 5 µL of total protein fractions after induction of SPPV-060 (lane 1), SPPV-095 (lane 2), SPPV-117 (lane 3), SPPV-122 (lane 4), and purified sheep pox virus (lane 5). The sera from the rabbits immunized with recombinant proteins SPPV-060 (**A**); SPPV-095 (**B**); SPPV-117 (**C**); SPPV-122 (**D**); and normal rabbit serum (**E**) were used to develop the blots. M: Spectra Multicolor Broad Range Protein Ladder (Thermo Scientific, Waltham, MA, USA).

All immunodetection assays were conducted by Western blot analysis. Recombinant protein production in E. coli was confirmed using anti-His (C-term)-AP antibody (Figure 3A). The second staining band in Figure 3A, lane 6 (not asterisked) may be due to target protein degradation. The antigenic properties of SPPV-060, SPPV-095, SPPV-117, and SPPV-122 proteins were detected using antisera obtained from experimentally SPPV infected sheep (Figure 3B, lanes 1–4). The immunogenicity of tested proteins was determined using antibodies raised via rabbit immunization with purified recombinant proteins (Figure 4A–D). The results of these experiments showed that antibodies in both cases reacted with the recombinant SPPV proteins.

### 3.3. Neutralization Assays of Polyclonal Antibodies Raised to the Recombinant Proteins

**Figure 5:**
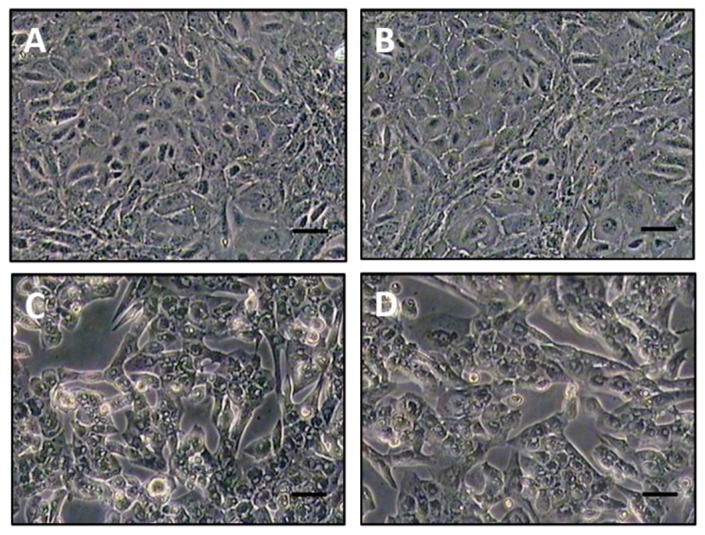
Monolayer of lamb kidney cell culture on the seventh day of incubation: (**A**) healthy cells (control-mock); (**B**) cells treated with a mixture of SPPV and antiserum raised to SPPV-117 protein in a dilution 1:2 (A, B; Magnification: 100×); (**C**) cells treated with a mixture of SPPV with antiserum raised to SPPV-117 protein in a dilution 1:32; (**D**) cells infected with SPPV (virus control). (C, D; Magnification: 200×).

The virus-neutralizing activities of antiserum raised to SPPV-060, SPPV-095, SPPV-117, and SPPV-122 proteins were investigated by monitoring CPE in monolayers of primary lamb kidney cell cultures infected with a viral suspension of SPPV (a mixture of EEV and IMV) treated with different antiserum dilutions (see Materials and Methods). The first signs of CPE appearance were observed in 48–72 h depending on the antiserum dilution and expressed through cell rounding, the appearance of cavities in the monolayer, cell detachment from vial walls, vacuolization of nuclei, loss of cellular monolayer continuity, and cytoplasmic inclusion body formation in the virus-affected cells (Figure 5D). The cytopathic effect was assessed on the seventh day post incubation. The wells exhibiting CPE (Figure 5C,D) were scored as “+” and the wells with CPE (Figure 5A,B) were scored as “-“. The results are shown in Table 2. The serum dilution that inhibited the virus-induced CPE in 50% of infected cell culture was considered as the titer of virus neutralizing antibodies. Antibodies to SPPV-060, SPPV-117 and SPPV-122 proteins neutralized SPPV with a serum titer of 3.75 log_2_, 4.0 log_2_, and 3.5 log_2_, respectively, however, antiserum to SPPV-095 protein failed to neutralize SPPV in cell culture (Table 2). Visual evidence for virus neutralization using a 1:2 dilution of SPPV-117 antiserum is shown in Figure 5B where the treated cells resemble healthy, untreated cells (Figure 5A) while cells treated with a mixture of SPPV and a 1:32 dilution of SPPV-117 antiserum show signs of CPE (Figure 5C), similar to those of virus-infected cells (Figure 5D). The “pre-bleed” sera were also analyzed in the virus neutralization test. Inhibition of virus-induced CPE in cell culture was not observed (data not shown).

## 4. Discussion

The choice of proteins used in this study was based on reports of the immunogenic properties of proteins of the most investigated representative of the family *Poxviridae*, vaccinia virus (VACV). According to the literature, some VACV proteins induce specific humoral and cellular immune responses and provide protection against subsequent orthopoxvirus infection. The most suitable candidates for subunit vaccine development are proteins encoded by A27L [45,46], D8L [47], H3L [48,49], L1R [21,50,51,52], B5R [53,54], A33R [55,56], and L4R and A4L [57] genes.

We investigated the antigenic and immunogenic properties of SPPV-060, SPPV-095, SPPV-117, and SPPV-122 proteins of SPPV, orthologs of VACV L1, A4, A27, and A33 proteins, respectively. To engineer the protein expression cassettes, amino acid sequence analysis of SPPV-060, SPPV-095, SPPV-117, and SPPV-122 proteins was conducted, which revealed that SPPV-060 and SPPV-122 possess putative transmembrane domains (data not shown). The presence of transmembrane domains and signal peptides in the target protein sequence can often result in either a lower level or absence of gene expression in bacterial cells [58]. In our experiments, in order to increase SPPV-060 and SPPV-122 production in *E. coli*, the gene sequences encoding the extravirion domains of these proteins were used to construct corresponding expression cassettes. The amplified SPPV regions encoding SPPV-060, SPPV-095, SPPV-117, and SPPV-122 proteins were cloned into the respective pET vectors (see Materials and Methods) and expressed in bacterial cells as N- or C-terminal His-tagged fusion proteins. One disadvantage of prokaryotic expression systems is the absence of post-translational modifications and, often, proper folding of proteins produced in bacterial cells [59]. However, it was shown by Berhanu *et al.* [60] that bacterially expressed A27L, B5R, and D8L proteins of VACV retained their antigenic and immunogenic properties. In our study, Western blot analysis revealed the reaction of bacterially-produced SPPV-060, SPPV-095, SPPV-117, and SPPV-122 proteins with antiserum from sheep experimentally infected animals with SPPV (Figure 3B, lanes 1–4) and with antiserum raised to purified recombinant proteins (Figure 4A–D). The results of these experiments confirm the antigenic and immunogenic properties of the recombinant proteins. It is interesting that sera of healthy animals revealed insignificant antibody binding to the recombinant proteins (Figure 3C, lanes 1–4). The slight immune background is probably associated with circulation of the virus among sheep as a result of using the live attenuated sheep pox vaccine in Kazakhstan. 

Recombinant SPPV-122 protein, an EEV envelope component, displayed antigenic and immunogenic properties that were confirmed by Western blot analysis. Moreover, rabbit immunization with SPPV-122 led to production of antibodies possessing virus-neutralizing activity towards SPPV. Interestingly, according to the literature, antibodies to VACV A33 protein, an ortholog of SPPV-122, did not neutralize VACV *in vitro* [61].

SPPV-060 and SPPV-117 proteins, components of the IMV envelope, possessed antigenic and immunogenic properties as well. They induced formation of virus-neutralizing antibodies (Table 2) as do their VACV orthologs (L1 and A27, respectively). Rodriguez *et al.* [62] reported that monoclonal antibodies to A27 protein completely inhibited virus-cell binding. Lai *et al.* [63] and Demkowicz *et al.* [57] demonstrated that immunization of mice with VACV A27 protein resulted in the production of virus-neutralizing antibodies in a high titer and the animals were completely protected against subsequent infection with a lethal dose of the VACV strain WR. VACV L1 protein induced production of IMV-neutralizing antibodies in mice in titers sufficient for complete protection against 5 LD50 of VACV WR [64], however at a higher infecting dose (100 LD50), only 28% of animals survived. In an independent study, it was shown that partial protection of mice against VACV infection was induced by polyclonal antiserum to L1 protein by passive immunization [65,66]. Moreover, L1 protein, a component of the mature virion membrane, is conserved in all sequenced poxviruses [67], so we can speculate that an L1-based vaccine developed against one poxvirus would stimulate immunity to the entire group of poxviruses [68]. The cross-neutralizing activity of antiserum—raised to soluble antigens—between Capripoxvirus members has also been demonstrated [69]. The conserved nature of ORF 117 (SPPV-117) between SPPV and GTPV isolates suggests that recombinant vaccine based on SPPV-117 may serve as a single vaccine for both diseases [70].

The high antigenicity of the SPPV-095 protein, found in the core of the sheep pox virion, and its potential use in sheep pox diagnosis via ELISA, has been reported by a number of authors [40,71]. Immunization of mice with VACV A4 protein, an ortholog of SPPV-095, induced a strong humoral immune response and ensured 50% protection against a lethal dose (40 LD50) of VACV [57]. In contrast, our results revealed that although the recombinant SPPV-095 recombinant protein was antigenic in experimental rabbits, the antibodies produced to this protein failed to neutralize SPPV in kidney cell culture. The role of SPPV-095 protein in modulation of the immune system is still a matter of discussion.

## 5. Conclusions

In summary, antibodies raised to bacterially-produced SPPV-060, SPPV-117, and SPPV-122 proteins neutralized SPPV in cell culture, while antibodies to SPPV-095 protein did not possess virus-neutralizing activity. The findings of our studies show that bacterially-produced SPPV-060, SPPV-117, and SPPV-122 proteins may be used for development efficient strategies for prevention of infectious diseases caused by members of *Capripoxvirus* genus, family *Poxviridae*. In the future, the efficacy of novel recombinant vaccines based on a combination of these proteins will be evaluated in large animal studies for their ability to protect sheep against sheep pox virus.

## Figures and Tables

**Table 1:** Oligonucleotide primers utilized in this study.

Primer Name	5′ → 3′ Sequence ^a^	Gene Amplified	Restriction Site
SPPV117-REV	gcatctcgagtcactttagtgttgtaattcttcctgttt	SPPV117 (NP_659689)	*Xho*I
SPPV117-DIR	gcatcat*atg*gacagagagcgttatcaatctttccaggcga		*Nde*I
SPPV095-DIR	cccat*atg*gacttcatgaaaaaaatatac	SPPV095 (NP_659667)	*Nde*I
SPPV095-REV	gcggccgctttgctgttattatcatcc		*Not*I
SPPV122-DIR	cccat*atg*catcatcatcatcatcataataatacatgtgaattaaatc	SPPV122∆TM (NP_659694)	*Xho*I
SPPV122-REV	ccctcgagttattaaaagttcatcatgaaaaaaagatcttacacagtaata		*Nde*I
SPPV060-DIR	attcat*atg*gagcagccgctagtatacaaac	SPPV060∆TM (NP_659632)	*Sac*I
SPPV060-REV	ctgcgagctctatataaaattgatatccgtatc		*Nde*I

^a^ Restriction sites are underlined; ATG start codon is indicated in italics.

**Table 2:** Sheep pox virus (SPPV) neutralization with the specific sera to the recombinant proteins.

Components	Serum Dilutions						Control			Geometric Average Titer of Neutralizing Antibodies Log_2_
	1:2	1:4	1:8	1:16	1:32	1:64	Virus (100 TCD_50_) + Medium	Serum 1:2 + Medium	medium	
Serum to the protein SPPV060 + virus (100 TCD_50_)	- - - -	- - - -	- - - -	- + + +	+ + + +	+ + + +	+ + + +	- - - -	- - - -	3.75
Serum to the protein SPPV095 + virus (100 TCD_50_)	+ + + +	+ + + +	+ + + +	+ + + +	+ + + +	+ + + +	+ + + +	- - - -	- - - -	0.0
Serum to the protein SPPV117 + virus (100 TCD_50_)	- - - -	- - - -	- - - -	- - + +	+ + + +	+ + + +	+ + + +	- - - -	- - - -	4.0
Serum to the protein SPPV122 + virus (100 TCD_50_)	- - - -	- - - -	- - - -	+ + + +	+ + + +	+ + + +	+ + + +	- - - -	- - - -	3.5

“+”, the presence of CPE in well; “-“, the absence of CPE in well.
